# Healthcare Workers’ Perspectives on the Upcoming COVID-19 Vaccine in Terms of Their Exposure to the Influenza Vaccine in Riyadh, Saudi Arabia: A Cross-Sectional Study

**DOI:** 10.3390/vaccines9050465

**Published:** 2021-05-06

**Authors:** Leena R. Baghdadi, Shatha G. Alghaihb, Alanoud A. Abuhaimed, Dania M. Alkelabi, Rawan S. Alqahtani

**Affiliations:** 1 Department of Family and Community Medicine, College of Medicine, King Saud University, Riyadh 11362, Saudi Arabia; 2 College of Medicine, King Saud University, Riyadh 11362, Saudi Arabia; 436200412@student.ksu.edu.sa (S.G.A.); 436202916@student.ksu.edu.sa (A.A.A.); 436200669@student.ksu.edu.sa (D.M.A.); 436200252@student.ksu.edu.sa (R.S.A.)

**Keywords:** COVID-19, pandemic, SARS-CoV-2, vaccine, acceptance

## Abstract

In 2019, a novel severe acute respiratory syndrome (SARS-CoV-2 (COVID-19)) caused a global pandemic. There was an urgent need to develop a vaccine against COVID-19 to reduce its spread and economic burden. The main objective of this study was to understand the attitudes and concerns of healthcare workers (HCWs) towards the upcoming COVID-19 vaccine, whether their decision was influenced by their history of taking the seasonal influenza vaccine, and factors that influence the acceptance of the upcoming COVID-19 vaccine. This was a cross-sectional study conducted in Riyadh, Saudi Arabia. We selected and surveyed 356 HCWs via an electronic self-administered questionnaire. A total of 61.16% of HCWs were willing to receive the COVID-19 vaccine, and 55.9% of them had received the seasonal influenza vaccine in the preceding year (2019–2020). The strongest predictors for taking the COVID-19 vaccine were the HCWs’ belief that the COVID-19 vaccine would be safe, needed even for healthy people, that all HCWs should be vaccinated against COVID-19, and that HCWs will have time to take the vaccine. Being female, being middle aged, having <5 years of work experience, having no fear of injections, and being a non-smoker were predictive factors for taking the upcoming COVID-19 vaccine. No associations were found between the intention to take the COVID-19 vaccine and a history of taking the seasonal influenza vaccine.

## 1. Introduction

In early 2020, a novel strain of the coronavirus, SARS-CoV-2 (COVID-19), spread rapidly over the world, and the World Health Organization (WHO) declared a global pandemic in March 2020. COVID-19 is caused by severe acute respiratory syndrome (SARS-CoV-2) [1]. The ongoing pandemic caused >98,956,091 confirmed cases and >2,121,638 deaths globally, as of 23 January 2021 [2]. The estimated Saudi population is 34,218,169 [3]; in Saudi Arabia, the first case was reported on 2 March 2020, and by 26 February 2021, the count increased to 376,723 confirmed cases and 6483 reported deaths [2,4].

The current global strategy to control the COVID-19 pandemic consists of suppression measures, including travel restrictions, quarantine, social distancing, case tracing, and case identification to prevent the spread of the infection [1,5]. Globally, the exponential spread of COVID-19 was a critical challenge for all nations. Saudi Arabia has been part of a previous pandemic, the Middle East respiratory syndrome coronavirus (MERS-CoV) in 2012. This experience of MERS-CoV helped the country implement strict measures during the COVID-19 pandemic, which mitigated its impact across Saudi Arabia in comparison to other countries [6]. Saudi Arabia was among the first countries to implement early, unprecedented, and strict precautionary measures to curb the spread of the pandemic and make a concerted effort to minimize the devastating effects of COVID-19 while the number of confirmed cases was <300 [7]. These measures included the activation of digital health, a national committee to follow global updates, implementation of a mandatory quarantine, remote learning, and suspension of all international Umrah pilgrimages. For the first time in Saudi Arabia’s history, mosques were closed nationwide, and Muslims were requested to pray at home [7].

Vaccines are the most effective measure to control infectious diseases and reduce the economic burden [8]. In 2009, the world responded to the pandemic H1N1 influenza (pH1N1). The strongest independent predictor of the intention to receive the pH1N1 vaccine was the receipt of the previous year’s seasonal influenza vaccine (odds ratio (OR) 6.25, 95% confidence interval (CI) 5.39–7.26) [9].

Candidate vaccines included an inactivated virus, nucleic acid, adenovirus base, and recombinant subunit vaccines [10]. In 2019, the WHO identified vaccine hesitancy as one of the top 10 global health threats [11]. The acceptance of new COVID-19 vaccines will be challenging and influenced by several determinants. Previous studies conducted on the general population of the United States, Italy, France, and China found that the acceptance levels of a COVID-19 vaccine were 69%, 86.1%, 77.6%, and 91.3%, respectively [12,13,14,15]. Many perceived factors influenced the acceptance of the COVID-19 vaccine, including being male, older age, fear about COVID-19, being a healthcare worker (HCW), and being at high risk of infection [14]. A study reported that being married, being vaccinated against influenza in the past season, believing in the efficacy of the COVID-19 vaccination, or valuing a doctor’s recommendations could increase the probability of accepting the COVID-19 vaccination [15]. Findings from a global survey, conducted to assess the potential acceptance of a COVID-19 vaccine across 19 countries, revealed heterogeneity in the responses. Differences in vaccine acceptance rates range from the highest in China (88.6%) to the lowest in Russia (54.9%) [16]. A recent community-based study conducted in Saudi Arabia found that 64.7% of people intended to take the COVID-19 vaccine, while only 7% reported hesitancy towards it. The willingness to accept the future COVID-19 vaccine was relatively high among participants who had a high education level (postgraduate or higher degrees) (68.8%), were non-Saudis (69.1%), and employed in the government sector (68.9%) [17].

HCWs are great public influencers. Therefore, a study of their attitudes and concerns towards the COVID-19 vaccine is an accurate predictor of the general population’s acceptance of the upcoming vaccine [18]. Congo’s HCWs revealed a 27.7% acceptance of the COVID-19 vaccine [19]. From HCWs in Los Angeles, 49.9% of them “would prefer to wait and first see how the vaccine affects others”, and 32.3% of HCWs reported their intention to get a coronavirus vaccine as soon as possible [20]. Another study showed a discrepancy in the acceptance of the COVID-19 vaccine among doctors (78%), nurses (61%, *p* < 0.01), and 75% of the general population [21]. A recent unpublished study of HCWs in Saudi Arabia reported that 70% of them were willing to receive a COVID-19 vaccine once available [22]. Thus, we hypothesize that the majority of HCWs will be willing to receive the COVID-19 vaccine on the basis of their history of receiving the seasonal influenza vaccine in the preceding year (2019–2020), and that vaccine uptake is influenced by their attitude and belief about its safety.

The main objective of this study was to understand the attitudes and concerns of HCWs towards the upcoming COVID-19 vaccine and whether their intention was affected by their history of taking the seasonal influenza vaccine. The secondary objective was to study the factors influencing the acceptance of the upcoming vaccine.

## 2. Materials and Methods

### 2.1. Study Population and Design

This was an analytical cross-sectional observational study. The target population was HCWs in the main hospitals of Riyadh, the capital of Saudi Arabia. Riyadh had one of the highest rates of confirmed COVID-19 cases in Saudi Arabia [23]. Data for the present study was collected between July and September 2020, when the COVID-19 vaccine was not available. English-speaking Saudi and non-Saudi HCWs working in Riyadh were included in the study. Those who were not HCWs, worked outside Riyadh, or had a known allergy to the vaccine components were excluded from the study. The study participants were selected via a convenience sampling technique. An online self-administered questionnaire was distributed via social media platforms, including LinkedIn, Twitter, Facebook, and WhatsApp, using a convenience sampling technique. The non-probability sampling technique was selected because the whole nation was under community-containment measures and asked to remain home, except for necessary trips. The survey was sent to the HCWs using SurveyMonkey©. The participants received reminder messages, which improved the response rate. Ethical approval was obtained from the Institutional Review Board (IRB) of the College of Medicine and King Saud University Medical City (approval no. E-20-4959). Before participation, all potential participants were provided with the purpose of the study and the opportunity to contact the principal investigator for any clarifications before signing online consent (Appendix A). Participation was voluntary, responses were anonymous, participant details were kept confidential, and the information was used only for data analysis.

### 2.2. Data Collection

A validated questionnaire (Appendix B), which can be accessed for academic and research purposes [24,25], was used. Permission was obtained from the authors before distributing the questionnaire to eligible participants. The survey begins with an online consent form stating all the responses will remain anonymous and confidential. After giving consent, the participants completed the questionnaire, which required ≤5 min. The questionnaire has three sections: demographic characteristics (including risk factors for a COVID-19 infection), seasonal influenza vaccination history, and factors influencing the intention to accept the COVID-19 vaccine. The factors thought to influence the participants’ intentions to accept the upcoming COVID-19 vaccine were assessed by 34 attitude-scale items, which have been validated and used by several studies [24,25]. The participants responded to questions or phrases designed to assess the common factors influencing their attitude and concerns about the COVID-19 vaccine on a 2-point Thurstone scale [24,25]. This scale surveys the five constructs of the Health Belief Model under six titles: perceived susceptibility to COVID-19, perceived severity of COVID-19, perceived benefits of the vaccination in preventing COVID-19, perceived barriers to accepting the vaccination, cues to action, and general attitudes [24,25]. A pilot study was conducted on 13 participants to assess the validity, comprehensibility, and the time required to complete the questionnaire. Responses from the pilot study were not included in the analysis of this paper. Data will be shared upon request, following ethical guidelines.

### 2.3. Power Sample Size and Statistical Analyses

Data were analyzed using Statistical Package for the Social Sciences (SPSS) version 27.0 (IBM Corporation, New York, NY, USA). The sample-size estimate was based on a previous study about respondents who intended to receive the pH1N1 vaccine [9]. Therefore, it was assumed that 69% of respondents will choose to receive the upcoming COVID-19 vaccine with a 5% margin of error and a 95% confidence interval (CI). Thus, the minimal sample size required was 329 participants. However, the final estimation for the sample size was 411, assuming a 20% non-response rate. *p*-values < 0.05 were considered statistically significant and a 95% CI was considered for the result. A chi-squared test was used to compare the variables and the willingness to accept the COVID-19 vaccine.

Using the COVID-19 Vaccine Attitudes Scale (34 items) (Appendix B), univariate analysis examined the differences between the participants willing and unwilling to accept the COVID-19 vaccine. The odds ratio, a 95% CI, and the *p*-value were calculated. Multivariate logistic regression analysis was used to predict the factors influencing acceptance of the COVID-19 vaccine among HCWs. Two independent regression models were generated to assess the ability of the attitude-scale items to predict the HCWs’ willingness to accept the COVID-19 vaccine. All factors found to be statistically significant (*p* < 0.05) were included in the final models. The base model included all the significant factors from the sociodemographic and influenza vaccine-history sections; however, it excluded the COVID-19 attitude scale items. The base model and the COVID-19 attitude scale items include the same factors from the base model, in addition to the significant items from the COVID-19 attitude scale. The area under the receiver operating curve (AUC) was used to assess the discriminative power of both models to accurately predict the HCWs’ COVID-19 vaccination decision.

## 3. Results

### 3.1. Subject Characteristics

Only 329/411 (80%) participants completed the entire questionnaire, and 363/411 (88.3%) participants answered the question about willingness to accept the upcoming COVID-19 vaccine. Overall, 222 (61.2%) HCWs were willing to accept the COVID-19 vaccine, with almost equal agreement between men (48.2%) and women (51.8%). The participants willing to accept the COVID-19 vaccine were aged 30–49 years (51.4%) and had <5 years work experience (41.0%). The participants willing to accept the vaccine were those with no fear of injections (70.7%) and no history of smoking (85.6%). The characteristics of the participants are presented in Table 1.

No statistically significant relationships were found between the willingness to accept the upcoming COVID-19 vaccine and nationality, social status, history of chronic diseases, household members (living with children, pregnant women, someone with a chronic illness), having close contact with an elderly person, pregnancy or breastfeeding, location, occupation, subspecialty, work status, having direct contact with patients in general, and dealing with confirmed COVID-19 patients. The least statistically significant characteristics were subspecialty, occupation, being pregnant, and a history of chronic diseases (Table 1). No statistically significant results were found between the participants’ intake of medications (including angiotensin-converting enzyme inhibitors, angiotensin II receptor blockers, immunosuppressants, and antirheumatic medications) and the willingness to accept the vaccination.

### 3.2. History of Taking the Seasonal Influenza Vaccine and COVID-19 Infection

The last seasonal influenza vaccine (2019–2020) was taken by 199 (55%) participants; only 56 (15.4%) participants took the seasonal influenza vaccine annually, while 68 (18.7%) participants had not taken the seasonal influenza vaccine in the past five years. The majority of study participants did not experience adverse effects 230/363 (63.3%) or allergies 308/363 (84.8%) from the seasonal influenza vaccine.

From the participants willing to accept the upcoming COVID-19 vaccine, 55.9% had taken the seasonal influenza vaccine in the preceding year (2019–2020). None of the items assessing the history of the seasonal influenza vaccine and COVID-19 infection showed a statistically significant relationship with the willingness to take the upcoming COVID-19 vaccine (Table 1).

### 3.3. Predictors of Healthcare Workers’ Acceptance of the COVID-19 Vaccine

Overall, most of the factors in the COVID-19 vaccine-attitudes scale were statistically significant (*p* < 0.05) and showed higher odds in those willing to accept the upcoming vaccine (Table 2). When assessing the perceived susceptibility to the COVID-19 infection, we found that the odds of the belief that the vaccine is required for a healthy person are significantly greater among HCWs willing to accept the upcoming vaccine (OR 8.59, 95% CI 5.14–14.37). Vaccine acceptors had greater odds about the belief that COVID-19 is a bad disease (OR 2.81, 95% CI 1.49–5.29) and is dangerous for patients (OR 2.79, 95% CI 1.42–5.5). Vaccine acceptors believed that the vaccine would be beneficial, as it is safe (OR 9.69, 95% CI 5.7–816.24) and would prevent the spread of COVID-19 (OR 8.99, 95% CI 4.99–16.19). Finding time to take the vaccine was not an obstacle for the acceptors (OR 18.03, 95% CI 8.1–839.78). Encouragement from close family and friends, colleagues, and supervisors influenced the HCWs’ decision to accept the vaccine (OR 7.42, 95% CI 4.32–12.74; OR 6.74, 95% CI 3.92–11.57; OR 6.29, 95% CI 3.62–10.92, respectively). The strongest predictive factor for vaccine acceptance was the belief that all HCWs should be vaccinated against COVID-19 (OR 29.53, 95% CI 12.86–67.81). In contrast, the vaccine rejectors believed that HCWs must have the freedom of choice (OR 0.42, 95% CI 0.23–0.77) to accept or reject the vaccine (Table 2).

Table 3 shows the factors included in the final multivariate logistic regression models with their OR, 95% CI, and *p*-values. In the base model, the AUC was 0.68 (95% CI 0.62–0.74); therefore, the base model predicted 68% of HCWs will accept the COVID-19 vaccine.

The second model integrates the base model and the COVID-19 Vaccine Attitudes Scale to determine whether this integration will increase the ability for predicting the HCWs’ acceptance of the vaccine. As presented in Table 3, the AUC of the integrated model was 0.92 (95% CI 0.89–0.95). Therefore, combining the base model with the attitude scale increased the ability to predict HCWs’ COVID-19 vaccine acceptance to 92%.

## 4. Discussion

To the best of our knowledge, this is one of the few studies that has explored the attitudes, concerns, and predictive factors for COVID-19 vaccine uptake among HCWs in relation to their exposure to the seasonal influenza vaccine. There were 61.2% of HCWs willing to receive the COVID-19 vaccine. However, no associations were found between the intention to accept the COVID-19 vaccine and the history of receiving the seasonal influenza vaccine.

The strongest predictive factor for vaccine acceptance was the belief that all HCWs should be vaccinated against COVID-19. Vaccine acceptance was higher in women, non-smokers, participants aged 30–49 years, and those without chronic diseases. Sociodemographic factors can determine not only the HCWs’ attitude but can also influence receiving the COVID-19 vaccine [26]. HCWs and ethnic minority groups are at increased risk of the COVID-19 infection and adverse outcomes. In line with our findings, a recent British study found that ethnic minority HCWs, especially younger females, were negatively associated with the COVID-19 vaccine uptake [27]. The rate was similar for previous pandemics. For example, uptake of the influenza vaccination among HCWs in Italy remains low, especially among those with no comorbidities and aged <44 years [28].

However, these findings differ from another report, where the highest vaccine acceptance rates were in older HCWs and patients with chronic diseases [19]. As our participant recruitment was via social media, most of the respondents were 30–49 years old, and perhaps there was limited access to the older age groups. Our study found a statistically significant association between having <5 years work experience and vaccination acceptance. This could be because younger age groups represented the majority of our respondents (89.8%). Although smoking is considered a risk factor for COVID-19 severity [1], we found approximately 53% of smokers unwilling to accept the COVID-19 vaccination. This finding highlights the need for future research and awareness programs for smokers. From an epidemiological perspective, these sociodemographic factors are considered important social confounders to vaccine response, which should be adjusted in the analyses to minimize the probability of the social desirability bias [29].

Our findings suggest that employment within the different healthcare professions (being a physician, nurse, or others) does not significantly influence the respondents’ acceptance or rejection of the upcoming COVID-19 vaccine. However, consistent with previous studies, our findings showed greater vaccine hesitation among nurses than physicians [20]. A study shows the COVID-19 vaccine acceptance among doctors (78%) was significantly higher than nurses (61%), and the overall acceptance rate for the COVID-19 vaccine among physicians and nurses is lower than their acceptance rates of the seasonal influenza vaccination [21]. The nursing staff are more likely to refuse vaccines because of common misconceptions about adverse effects and efficacy [30]. Future research is needed to investigate this negative attitude, since nurses have close contact with patients.

A recent national unpublished study assessed the acceptance rate of the COVID-19 vaccine among HCWs [22] and reported that most HCWs (86%) had been exposed to patients with COVID-19. However, only 40% of our study participants had exposure to confirmed COVID-19 cases as our data was collected in July, while their data was collected in November, when the exposure to COVID-19 cases was higher. In addition, the HCWs’ attitude towards the upcoming vaccines was similar. They reported 70% of HCWs willing to accept the COVID-19 vaccine, compared to 61.2% of HCWs in our study [22].

Several studies indicate that a positive attitude towards the seasonal influenza vaccination is a strong determinant for accepting the COVID-19 vaccine [21,22]. Nevertheless, our study found no association between the COVID-19 vaccine acceptance and the seasonal influenza vaccine uptake. This can be attributed to several factors, including the fact that this study was a cross-sectional observational study. We found that our study participants’ uptake of the seasonal influenza vaccine in the past season (2019–2020) was suboptimal (55%), being consistent with previous national studies conducted in the cities of Arar and Abha, where the seasonal influenza vaccination (2017–2018) coverage rates among HCWs were 55.9% and 45.5%, respectively [31,32].

We expect that the current COVID-19 pandemic will promote a higher compliance of the seasonal influenza vaccines in HCWs, forming a bidirectional relationship [18,33]. The perception that COVID-19 was an “enhanced influenza” could contribute to the increased willingness in HCWs to take the seasonal influenza vaccine [18]. A study conducted in Italy assessing the willingness to take the seasonal influenza and COVID-19 vaccines in the three phases of the COVID-19 lockdown (pre-lockdown, lockdown, and re-opening) showed an increase in the number of adults planning to take the seasonal influenza vaccine in the lockdown-reopening phase [33].

COVID-19 vaccine is the only effective measure to confine the pandemic and eventually resume much of regular life, and HCWs have priority to be vaccinated. Our study found that the COVID-19 vaccine acceptance was related to the HCWs’ modifiable beliefs and attitudes that the COVID-19 vaccine is safe, needed even for healthy people, that all HCWs should be vaccinated against COVID-19, and that HCWs will have time to take the vaccine. Research on the pH1N1 vaccine showed findings similar to our research, where HCWs were more likely to accept the pH1N1 vaccination if they perceived a susceptibility to the infection, severity of the infection, the benefits of the vaccination outweighed the barriers, and HCWs were influenced by positive external cues to action [24].

The belief that all HCWs should be vaccinated against COVID-19 was the strongest predictive factor for vaccine uptake. This finding reflects the importance of HCWs to be vaccinated, as they are the first line of defense against COVID-19. HCWs have a critical role in promoting vaccine culture in their community. Therefore, the first step to vaccine acceptance is developing the confidence in HCWs in receiving the vaccine and recommending it to their patients [22].

The Saudi government linked completing the vaccines by individuals to one of the mega events held yearly in Makkah, “the Hajj”. During the current holy month (Ramadan), the Saudi Arabian Ministry of Hajj and Umrah allowed pilgrims into the Grand Mosque in Makkah if they could show evidence of immunity by providing a certificate of either having received at least one dose of the COVID-19 vaccine or having a history of a previous COVID-19 infection, and this was to be presented before entering the holy mosque, praying, or performing Umrah [34]. A health passport is required for overseas travel. In fact, many routine activities, including football matches, mandate receiving COVID-19 vaccination.

HCWs were in the first priority group for vaccination, and they were at the first stage of the Centers for Disease Control and Prevention (CDC) COVID-19 Vaccine Rollout Recommendation as they are the frontline in fighting the pandemic [35]. The findings from this study provide an insight about the attitude of HCWs and their willingness to take the COVID-19 vaccines in terms of their previous exposure to the influenza vaccination. This could aid the government in preparedness for future pandemics by initiating legislation and policies mandating vaccination for all HCWs, as seen with the previous pandemic (i.e., influenza vaccination), whereby all HCWs should receive the influenza vaccine annually [36]. A recent British study found that ethnic minority HCWs, especially the younger females, were negatively associated with uptake of the COVID-19 vaccine [27]. Although challengers argue that HCWs have the right to refuse the vaccination and wear personal protective equipment (PPE) instead, a recent paper analyzing the law and policy of Canadian vaccination for HCWs recommended that governments should put in place policies for mandatory vaccination of all HCWs to reduce the overall burden of COVID-19 [37].

This study has numerous strengths. It was conducted in Riyadh, the capital and largest city in Saudi Arabia. HCWs from multiple centers were enrolled in the study, including government hospitals, university hospitals, and private hospitals. An adequate sample size was achieved with a good response rate, despite being an online survey. Despite having an adequate sample size, we had expected a higher response rate. Online surveys are usually associated with low response rate compared with face-to face survey. This was expected especially true during the COVID-19 pandemic as many people, especially HCWs, experienced higher psychological stress, in addition to the increased number of online surveys leading to survey fatigue.

This is one of the few studies that has assessed the association between the influenza vaccine status and the rate of the COVID-19 vaccine acceptance, measured the HCWs’ willingness to take the COVID-19 vaccine using an attitude scale, and tested it by multivariate logistic regression.

However, the single geographical location may limit its generalizability. Data were collected before the availability of a vaccine, which could influence the HCWs’ decisions. As it was an online questionnaire open to all HCWs, it was difficult to have an equal distribution of age, occupation, and specialties in the participants.

Our results recommend that the HCW attitudes and concerns towards COVID-19 should be addressed. The opinion of HCWs has high value in communities; therefore, it is essential to target HCWs with awareness campaigns to change their perceptions. Future vaccine campaigns will have a greater impact in pandemic and non-pandemic times if vaccine culture is promoted in the workplace among colleagues with the supervisor’s encouragement. The pandemic lockdown caused some difficulties in reaching the targeted sample size; therefore, we recommend establishing an electronic network to connect HCWs from different facilities and specialties. We suggest conducting further studies to assess the COVID-19 vaccine uptake after the vaccine is available. National longitudinal studies are required to assess the attitudes of HCWs towards influenza vaccines using the Vaccine Attitude Scale, as well as to assess the influenza vaccine uptake after the COVID-19 pandemic.

## 5. Conclusions

In conclusion, most of the HCWs were willing to accept the COVID-19 vaccine. The intention of accepting the COVID-19 vaccine was not associated with previous exposure to the seasonal influenza vaccine. The modifiable factors, which were the strongest predictors for taking the COVID-19 vaccine, were the HCWs’ beliefs that the COVID-19 vaccine will be safe, needed even if the person is healthy, that all HCWs should be vaccinated against COVID-19, and that the HCWs will have time to take the vaccine.

## Figures and Tables

**Table 1:** Sociodemographic factors and influenza vaccination history as predictors of the healthcare workers’ COVID-19 vaccine acceptance.

Characteristics		I Am Willing to Accept the Upcoming COVID-19 Vaccine		*p*-Value
		No (*n* = 141)	Yes (*n* = 222)	
Sociodemographic				
Age (years)	<30	27.7%	40.5%	0.025
	30–49	58.9%	51.4%	
	≥50	13.5%	8.1%	
Gender	Men	38.3%	48.2%	0.040
	Women	61.7%	51.8%	
Nationality	Saudi	70.2%	74.8%	0.202
	Non-Saudi	29.8%	25.2%	
Social status	Single	38.3%	41.9%	0.320
	Married	60.3%	54.5%	
	Divorced or widowed	1.4%	3.6%	
History of chronic diseases	No	80.1%	80.2%	0.548
	Yes	19.9%	19.8%	
I live with children	No	36.2%	44.6%	0.069
	Yes	63.8%	55.4%	
I live with a pregnant woman	No	97.2%	95.5%	0.306
	Yes	2.8%	4.5%	
I live with someone who has a chronic illness	No	64.5%	61.7%	0.334
	Yes	35.5%	38.3%	
Outside work, I have close contact with an elderly person	No	45.4%	42.8%	0.353
	Yes	54.6%	57.2%	
I have fear of injections	Not at all	63.8%	70.7%	0.044
	Yes, I try to avoid injections	12.8%	5.4%	
	I have some fear; I do not avoid injections	23.4%	23.9%	
I am a smoker	No	74.5%	85.6%	0.006
	Yes	25.5%	14.4%	
I am pregnant	No	97.7%	97.4%	0.630
	Yes	2.3%	2.6%	
I am breastfeeding	No	98.9%	96.5%	0.280
	Yes	1.1%	3.5%	
Occupation details				
Location	Riyadh (main city)	97.9%	98.6%	0.432
	Riyadh (villages and provinces)	2.1%	1.4%	
Occupation	Physician	42.6%	44.6%	0.888
	Nurse	21.3%	19.4%	
	Others	36.2%	36.0%	
Subspecialty	Emergency	7.1%	7.2%	0.991
	Intensive care unit	8.5%	8.6%	
	Medicine	7.1%	7.7%	
	Surgery	8.5%	8.1%	
	Family Medicine	9.2%	7.2%	
	Other	59.6%	61.3%	
Work status	Full-time	97.2%	96.4%	0.470
	Part-time	2.8%	3.6%	
Years of work experience	<5 years	27.7%	41.0%	0.033
	5–10 years	27.0%	23.4%	
	>10 years	45.4%	35.6%	
Work institution	King Khalid University Hospital	16.3%	12.6%	0.734
	King Abdulaziz University Hospital	3.5%	3.6%	
	National Guard Health Affairs	7.8%	9.5%	
	Prince Sultan Military Medical City	17.0%	13.1%	
	King Saud Medical City	2.8%	6.3%	
	Security Forces Hospital in Riyadh	4.3%	4.5%	
	King Faisal Specialist Hospital and Research Center	7.8%	6.8%	
	King Fahad Medical City	3.5%	5.9%	
	Others	36.9%	37.8%	
In my work, I have direct contact with patients	No	22.7%	15.3%	0.052
	Yes	77.3%	84.7%	
I deal with confirmed COVID-19 patients	No	62.4%	59.0%	0.297
	Yes	37.6%	41.0%	
History of seasonal influenza vaccine				
I received the last seasonal influenza vaccine (2019–2020)	No	46.8%	44.1%	0.348
	Yes	53.2%	55.9%	
In the past five years, how many times did you take seasonal influenza vaccine?	Did not take the vaccine	24.1%	15.8%	0.229
	1–2 times	38.3%	45.0%	
	3–4 times	22.0%	24.3%	
	Every year	15.6%	14.9%	
In the past, I had adverse effects from the seasonal influenza vaccine:	No	66.0%	61.7%	0.214
	Yes	15.6%	23.0%	
	Not sure	18.4%	15.3%	
I am allergic to the seasonal influenza vaccine components	No	80.1%	87.8%	0.089
	Yes	1.4%	1.8%	
	Not sure	18.4%	10.4%	
History of COVID-19 infection				
I had COVID-19 infection	No	92.2%	94.6%	0.242
	Yes	7.8%	5.4%	
One of my family members or friends got COVID-19 infection	No	60.3%	52.7%	0.095
	Yes	39.7%	47.3%	

**Table 2:** Healthcare workers’ COVID-19 Vaccine Attitudes Scale items as predictors of accepting the COVID-19 vaccine.

Willing to Accept the Upcoming COVID-19’. Then under It, No and Yes; and OR and *p*-Value	Willing to Accept the Upcoming COVID-19 Vaccine (*n* = 329)		Heading	Heading
Factor	No (*n* = 125)	Yes (*n* = 204)	OR ^a^ (95% CI ^b^)	*p*-Value *
Perceived susceptibility to COVID-19 disease:				
1—I am at high personal risk for getting COVID-19	21.6%	42.2%	1.63 (1.03–2.58)	0.025 *
2—It is very likely that I can infect patients with COVID-19 if I DON’T get the SARS-CoV-2 (COVID-19) vaccine	10.6%	39.2%	4.42 (2.73–7.17)	<0.001 *
3—I am likely to get COVID-19 if I DON’T get the SARS-CoV-2 (COVID-19) vaccine	14.9%	41.3%	3.1 (1.95–4.92)	<0.001 *
4—The SARS-CoV-2 (COVID-19) vaccine is required for a healthy person	13.4%	51.1%	8.59 (5.14–14.37)	<0.001 *
5—Healthcare workers are at greater risk than general public of catching COVID-19	33.1%	60.2%	4.84 (1.84–12.74)	0.001 *
6—I am at risk of catching COVID-19 from hospital patients	28.9%	58.1%	4.64 (2.31–9.3)	<0.001 *
Perceived severity of COVID-19 disease				
7—COVID-19 is dangerous for the patients in the hospital at which I work	30.7%	57.1%	2.79 (1.42–5.5)	0.002 *
8—COVID-19 is a bad disease	29.5%	56.2%	2.81 (1.49–5.29)	0.001 *
9—If I were to get COVID-19, it would significantly interfere with my regular daily activities	30.4%	53.5%	1.57 (0.87–2.84)	0.090
10—Other health problems that I have may become worse if I get COVID-19	24.3%	43.5%	1.32 (0.82–2.12)	0.152
11—The thought of getting COVID-19 scares me	18.8%	38.3%	1.64 (1.05–2.57)	0.020 *
Perceived benefits of vaccination in preventing COVID-19 disease				
12—If I get vaccinated against SARS-CoV-2 (COVID-19), then I will be more certain that I will not infect patients	16.1%	48.0%	4.67 (2.88–7.57)	<0.001 *
13—If I get vaccinated against SARS-CoV-2 (COVID-19), then I will be more certain that I will not infect family members	17.9%	50.8%	5.05 (3.06–8.33)	<0.001 *
14—Getting the SARS-CoV-2 (COVID-19) vaccine will prevent me from getting COVID-19	14.0%	46.8%	5.29 (3.26–8.58)	<0.001 *
15—Getting the SARS-CoV-2 (COVID-19) vaccine will prevent spread of COVID-19	19.8%	56.2%	8.99 (4.99–16.19)	<0.001 *
16—The SARS-CoV-2 (COVID-19) vaccine will NOT cause COVID-19	18.5%	44.7%	2.71 (1.7–4.31)	<0.001 *
17—I do not expect any side effects (e.g., local tenderness or infection) from the SARS-CoV-2 (COVID-19) vaccine	7.3%	18.5%	1.8 (1.05–3.07)	0.021 *
18—I do not expect an allergic reaction or autoimmune disease after getting the SARS-CoV-2 (COVID-19) vaccine	10.0%	27.7%	2.25 (1.38–3.64)	0.001 *
19—I believe the SARS-CoV-2 (COVID-19) vaccine will be safe	11.6%	50.2%	9.69 (5.78–16.24)	<0.001 *
Perceived barriers to accepting vaccination				
20—I will have time to get the SARS-CoV-2 (COVID-19) vaccine	21.9%	59.6%	18.03 (8.18–39.78)	<0.001 *
21—SARS-CoV-2 (COVID-19) vaccine will NOT be painful	20.1%	45.0%	2.36 (1.48–3.77)	<0.001 *
22—Getting the SARS-CoV-2 (COVID-19) vaccine will NOT interfere with my daily activities	23.4%	51.1%	2.91 (1.75–4.84)	<0.001 *
23—I am NOT worried about the side effects of getting the SARS-CoV-2 (COVID-19) vaccine	9.1%	36.8%	4.62 (2.81–7.58)	<0.001 *
24—The SARS-CoV-2 (COVID-19) vaccine will NOT make me sick	8.5%	36.2%	4.85 (2.93–8.03)	<0.001 *
Cues to action				
25—People close to me think that it is important for me to get vaccinated against COVID-19	18.2%	54.1%	7.42 (4.32–12.74)	<0.001 *
26—My colleagues think it is important for me to get the SARS-CoV-2 (COVID-19) vaccine	19.1%	54.1%	6.74 (3.92–11.57)	<0.001 *
27—My doctor encourages me to get the SARS-CoV-2 (COVID-19) vaccine	16.4%	48.3%	4.65 (2.86–7.54)	<0.001 *
28—My supervisors think it is a good idea for me to get the SARS-CoV-2 (COVID-19) vaccine	20.7%	54.7%	6.29 (3.62–10.92)	<0.001 *
General attitudes				
29—All healthcare workers should be vaccinated against COVID-19	18.5%	59.9%	29.53 (12.86–67.81)	<0.001 *
30—It is important that healthcare workers have freedom of choice in vaccination	33.1%	45.9%	0.42 (0.23–0.77)	0.003 *
31—I believe in immunizations	33.7%	60.8%	6.31 (2.03–19.62)	0.001 *

**^a^** OR, odds ratio; **^b^** CI, confidence interval. For the OR, the reference group = HCWs who were not willing to take the upcoming vaccine. * *p* ≤ 0.05 was considered statistically significant.

**Table 3:** Multivariate regression analysis: modeling factors predictive of healthcare workers’ acceptance of the COVID-19 vaccine.

Predictor Variables	Base Model ^a^			Base Model + Attitudes Scale Items ^b^		
	OR ^c^	95% CI ^d^	*p*-Value	OR ^c^	95% CI ^d^	*p*-Value
**Sociodemographic**						
Age						
<30 years	Reference	Reference	0.261	Reference	Reference	0.937
30–49 years	0.68	(0.33–1.40)	0.295	0.86	(0.27–2.77)	0.803
≥50 years	0.41	(0.14–1.19)	0.102	0.72	(0.12–4.35)	0.718
I have fear of injections						
Not at all	Reference	Reference	0.021	Reference	Reference	0.481
Yes, and I try to avoid injections as much as possible	0.32	(0.15–0.72)	0.006	0.45	(0.11–1.79)	0.255
I have some fear, however, I do not avoid injections	0.82	(0.48–1.38)	0.448	0.76	(0.33–1.76)	0.525
I am NOT a smoker	0.42	(0.24–0.73)	0.002	0.55	(0.22–1.35)	0.189
Years of work experience						
<5 years	Reference	Reference	0.694	Reference	Reference	.569
5–10 years	0.74	(0.35–1.52)	0.408	0.71	(0.22–2.31)	0.568
>10 years	0.84	(0.37–1.89)	0.673	0.50	(0.14–1.84)	0.297
**Covid-19 Vaccine Attitudes Scale items**						
1—I am at high personal risk for getting COVID-19				1.19	(0.55–2.58)	0.666
2—It is very likely that I can infect patients with COVID-19 if I DON’T get the SARS-CoV-2 (COVID-19) vaccine				2.58	(1.07–6.21)	0.035
3—I am likely to get COVID-19 if I DON’T get the SARS-CoV-2 (COVID-19) vaccine				0.56	(0.22–1.39)	0.207
4—The SARS-CoV-2 (COVID-19) vaccine is required for a healthy person				3.1	(1.70–9.4)	0.001
5—Healthcare workers are at greater risk than general public of catching COVID-19				1.03	(0.21–5.11)	0.970
6—I am at risk of catching COVID-19 from hospital patients				2.55	(0.74–8.76)	0.138
7—COVID-19 is dangerous for the patients in the hospital at which I work				1.08	(0.33–3.55)	0.900
8—COVID-19 is a bad disease				0.73	(0.24–2.23)	0.583
11—The thought of getting COVID-19 scares me				1.14	(0.53–2.47)	0.736
12—If I get vaccinated against SARS-CoV-2 (COVID-19), then I will be more certain that I will not infect patients				0.78	(0.25–2.44)	0.669
13—If I get vaccinated against SARS-CoV-2 (COVID-19), then I will be more certain that I will not infect family members				1.07	(0.31–3.69)	0.918
14—Getting the SARS-CoV-2 (COVID-19) vaccine will prevent me from getting COVID-19				0.64	(0.23–1.79)	0.393
15—Getting the SARS-CoV-2 (COVID-19) vaccine will prevent spread of COVID-19				2.16	(0.64–7.29)	0.214
16—The SARS-CoV-2 (COVID-19) vaccine will NOT cause COVID-19				0.38	(0.15–0.96)	0.040
17—I do not expect any side effects (eg, local tenderness or infection) from the SARS-CoV-2 (COVID-19) vaccine				0.45	(0.16–1.26)	0.126
18—I do not expect an allergic reaction or autoimmune disease after getting the SARS-CoV-2 (COVID-19) vaccine				0.76	(0.3–1.93)	0.560
19—I believe the SARS-CoV-2 (COVID-19) vaccine will be safe				4.36	(1.65–11.5)	0.003
20—I will have time to get the SARS-CoV-2 (COVID-19) vaccine				6.69	(2.21–20.2)	0.001
21—SARS-CoV-2 (COVID-19) vaccine will NOT be painful				1.24	(0.53–2.91)	0.616
22—Getting the SARS-CoV-2 (COVID-19) vaccine will NOT interfere with my daily activities				0.42	(0.14–1.21)	0.106
23—I am NOT worried about the side effects of getting the SARS-CoV-2 (COVID-19) vaccine				2.46	(1.01–5.98)	0.047
24—The SARS-CoV-2 (COVID-19) vaccine will NOT make me sick				1.62	(0.67–3.94)	0.289
25—People close to me think that it is important for me to get vaccinated against COVID-19				3.33	(1.0–11.1)	0.051
26—My colleagues think it is important for me to get the SARS-CoV-2 (COVID-19) vaccine				0.97	(0.24–3.88)	0.970
27—My doctor encourages me to get the SARS-CoV-2 (COVID-19) vaccine				0.23	(0.07–0.85)	0.028
28—My supervisors think it is a good idea for me to get the SARS-CoV-2 (COVID-19) vaccine				1.18	(0.31–4.49)	0.806
29—All healthcare workers should be vaccinated against COVID-19				9.02	(2.44–33.4)	0.001
30—It is important that healthcare workers have freedom of choice in vaccination				0.63	(0.23–1.71)	0.368
31—I believe in immunizations				3.43	(0.48–24.3)	0.218
**Area under the receiver operating curve**	0.64	(0.58–0.74)	<0.001	0.92	(0.88–0.95)	<0.001

^a^ Base model: includes factors from the sociodemographic section, which have *p* < 0.05, and excludes COVID-19 Vaccine Attitudes Scale items. ^b^ Base model plus COVID-19 Vaccine Attitudes Scale items: includes all factors in base model and the COVID-19 Vaccine Attitudes Scale items, which have *p* < 0.05. ^c^ OR, odds ratio; ^d^ CI, confidence interval.

## Data Availability

Data will be shared upon reasonable request and after approval from the institutional review board. A proposal with a detailed description of study objectives and a statistical analysis plan will be needed for the assessment of requests. Additional materials could be required during the process of assessment. De-identified participant data will be provided after approval by the investigators.

## Appendix A

Consent form

## Appendix B

Validated questionnaire

1- **Demographic characteristics.**
  **First: Personal Information**		
  **1- Age:** A. <30 B. 30–49 C ≥50	**2-Gender:** A. Male B. Female	**3-Nationality:** A. Saudi B. Non-Saudi, please specify: 1) Egyptian 2) Syrian 3) Filipino 4) Indian 5) Other, please specify:
  **4-Social status:** A. Single B. Married C. Divorced D. Widow	**5-Work city:** A. Riyadh B. Others	**6-I have a chronic illness:** A. Yes B. No If **yes**, please choose from the following: 1) Diabetes mellitus 2) Hypertension 3) Cardiovascular disease 4) Kidney disease 5) Respiratory disease 6) Rheumatic disease 7) Others, please specify:
  **7-Are you on ACE inhibitor (antihypertensive medication) such as enalapril (Vasotec) or lisinopril (Zestril)...?** A. Yes (please write down the medication name): B. No	**8-Are you on ARBs (antihypertensive medication) such as valsartan (Diovan) or azilsartan (Edarbi)?** A. Yes (please write down the medication name): B. No	**9-Are you on immunosuppressant medication such as azathioprine (Imuran) or methotrexate...?** A. Yes (please write down the medication name): B. No
  **10-Are you on anti-rheumatic medication such as hydroxychloroquine or methotrexate...?** A. Yes (please write down the medication name): B. No	**11-Are you on corticosteroid medication such as dexamethasone or prednisone...?** A. Yes (please write down the medication name): B. No	**12-For females: I am pregnant:** A. Yes B. No
  **13-For females:I am breastfeeding:** A. Yes B. No	**14-I live with children:** A. Yes B. No	**15-I live with a pregnant lady:** A. Yes B. No
  **16-I live with someone who has a chronic illness:** A. Yes B. No	**17-Outside work, I have close contact with an elderly person:** A. Yes B. No	**18-I have fear of injections:** A. Yes B. No
  **19-I am a smoker:** A. Yes B. No		
  **Second: Work information**		
  **1-Occupation:** A. Physician B. Nurse C. Other, please specify:...	**2-Work status:** A. Full time B. Part time	**3-Years of work experience:** A. < 5 years B. 5-10 years C. > 10 years
  **4-Work institution:** A. King Khalid University Hospital (KKUH) B. King Abdulaziz University Hospital (KAUH) C. National Guard Health Affairs (NGHA) D. Prince Sultan Military Medical City (PSMMC) E. King Saud Medical City (KSMC) F. Security Forces Hospital in Riyadh (SFH) G. King Faisal Specialist Hospital and Research Center (KFSHRC) H. King Fahad Medical City (KFMC) I. Other, please specify:...	**5-Subsector:** A. Emergency B. Intensive care unit C. Medicine D. Surgery E. Family Medicine F. Other, please specify:...	**6-In my work, I have direct contact with patients:** A. Yes B. No
  **7-I am dealing with confirmed COVID-19 patients:** A. Yes B. No		
2- **Seasonal influenza vaccine**
  **Seasonal Influenza Vaccination History**		
  **1-I received the last seasonal influenza vaccine (2019–2020):** A. Yes B. No	**2-In the past five years, how many times did you take the seasonal influenza vaccine?** A. I did NOT take the vaccine in the past five years B. 1–2 times C. 3–4 times D. I did take it in all the past five years	**3-In the past, I had side effects from seasonal influenza vaccine:** A. Yes B. No C. Not sure
  **4-I am allergic to seasonal influenza vaccine components:** A. Yes B. No C. Not sure		
3- **SARS-CoV-2 (COVID-19) vaccine**
  **First: History of COVID-19 Infection**				
  **Are you willing to take the upcoming SARS-CoV2 (COVID-19) vaccine**? Yes No	**Have you got COVID-19 infection**? Yes No	**Have any of your family members or friends got COVID-19 infection?** Yes No		
  **Second: Factors influencing intentions to accept pandemic SARS-CoV-2 vaccine.**				
  Please specify whether you agree or disagree with the following statements:				
  **Statement**			**Agree**	**Disagree**
  **Perceived susceptibility to COVID-19 disease:**				
  I am at high personal risk for getting COVID-19				
  It is very likely that I can infect patients with COVID-19 if I don’t get the SARS-CoV-2 (COVID-19) vaccine				
  I am likely to get COVID-19 if I do not get the SARS-CoV-2 (COVID-19) vaccine				
  The SARS-CoV-2 (COVID-19) vaccine IS required for a healthy person				
  Healthcare workers are at greater risk than general public of catching COVID-19				
  I am at risk of catching COVID-19 from hospital patients				
  **Perceived severity of COVID-19 disease**				
  COVID-19 is dangerous for me				
  COVID-19 is dangerous for the patients in the hospital at which I work				
  COVID-19 is a bad disease				
  If I were to get COVID-19, it would significantly interfere with my regular daily activities				
  Other health problems that I have may become worse if I get COVID-19				
  The thought of getting COVID-19 scares me				
  **Perceived benefits of vaccination in preventing COVID-19 disease**				
  If I get vaccinated against SARS-CoV-2 (COVID-19), then I will be more certain that I will not infect patients				
  If I get vaccinated against SARS-CoV-2 (COVID-19), then I will be more certain that I will not infect family members				
  Getting the SARS-CoV-2 (COVID-19) vaccine will prevent me from getting COVID-19				
  Getting the SARS-CoV-2 (COVID-19) vaccine will prevent spread of COVID-19				
  The SARS-CoV-2 (COVID-19) vaccine will NOT cause COVID-19				
  I do not expect any side effects (e.g., local tenderness or infection) from the SARS-CoV-2 (COVID-19) vaccine				
  I do not expect an allergic reaction or autoimmune disease after getting the SARS-CoV-2 (COVID-19) vaccine				
  I believe the SARS-CoV-2 (COVID-19) vaccine will be safe				
  **Perceived barriers to accepting vaccination**				
  I will have time to get the SARS-CoV-2 (COVID-19) vaccine				
  SARS-CoV-2 (COVID-19) vaccine will NOT be painful				
  Getting the SARS-CoV-2 (COVID-19) vaccine will NOT interfere with my daily activities				
  I am NOT worried about the side effects of getting the SARS-CoV-2 (COVID-19) vaccine				
  The SARS-CoV-2 (COVID-19) vaccine will NOT make me sick				
  **Cues to action**				
  People close to me think that it is important for me to get vaccinated against COVID-19				
  My colleagues think it is important for me to get the SARS-CoV-2 (COVID-19) vaccine				
  My doctor encouraged me to get the SARS-CoV-2 (COVID-19) vaccine				
  My supervisors thought it was a good idea for me to get the SARS-CoV-2 (COVID-19) vaccine				
  **General attitudes**				
  All healthcare workers should be vaccinated against COVID-19				
  It is important that healthcare workers have freedom of choice in vaccination				
  I believe in immunizations				
